# *Sambucus nigra* alleviates fenpropathrin-induced hepatorenal toxicity in rats via modulation of NF-κB/TNF-α axis

**DOI:** 10.1038/s41598-025-13653-5

**Published:** 2025-08-06

**Authors:** Adel F. Tohamy, Marwa H. Hassan, Abdelbary M. Prince, Marsail S. Nashed, Eman I. Hassanen, Maher M. Soliman

**Affiliations:** 1 Department of Toxicology and Forensic Medicine, Faculty of Veterinary Medicine, Cairo University, Giza, Egypt; 2 Department of Veterinary Clinical Sciences, Faculty of Veterinary Medicine, Jordan University of Science and Technology, Irbid, Jordan; 3 Department of Surgery, Anesthesiology and Radiology, Faculty of Veterinary Medicine, Cairo University, Giza, Egypt; 4 Department of Biochemistry and Molecular Biology, Faculty of Veterinary Medicine, Cairo University, Giza, Egypt; 5 Department of Pathology, Faculty of Veterinary Medicine, Cairo University, Giza, Egypt

**Keywords:** Cell death, Fenpropathrin, Inflammation, *Sambucus Nigra*, TNF-α, NF-κB, Biochemistry, Chemical biology, Plant sciences, Environmental sciences, Medical research, Nephrology

## Abstract

This research assessed the effectiveness of *Sambucus nigra* (SN) in alleviating hepatorenal injury caused by fenpropathrin (FNP) in rodents. Six equal groups were created from the 30 Wistar rats: Group 1 was the negative control, Groups 2 and 3 were the SN control groups, Group 4 was the FNP group, and Groups 5 and 6 were the FNP + SN combination groups. The hepatoprotective and renoprotective effects of SN were assessed by quantifying serum enzyme markers, including ALT, AST, ALP, blood urea nitrogen, and creatinine. Oxidative stress indicators, RT-PCR analysis, histological examination, and immunohistochemistry studies were conducted on the liver and kidneys to confirm the previously indicated parameters. The rats administered FNP injections displayed increased blood marker enzyme levels, altered oxidant-antioxidant equilibrium, and significant pathogenic changes in hepatic and renal tissues. Furthermore, these rats exhibited elevated levels of caspase-3 and iNOS, linked to the triggered expression of TNF-α and NF-κB genes in these tissues. Administering SN enhanced all the aforementioned toxicological parameters. The prospective hepato-renal therapeutic benefits of SN against impairment of the liver and kidneys induced by FNP have been evidenced through its anti-inflammatory, antioxidant, and anti-apoptotic pathways.

## Introduction

Ecological contamination constitutes a significant source of safety hazards worldwide, especially in agricultural countries, where poverty, insufficient investment in modern technology, and inadequate environmental legislation converge to produce elevated pollution levels. Prolonged exposure to pesticides is regarded as a contributing cause of several diseases in humans and livestock, notwithstanding the problem of ecological contamination^1^. Pesticides are utilized extensively in horticulture and veterinary care; nonetheless, they may adversely affect wildlife, humans, and domesticated animals^2^. Persistent pesticides can be conveyed between regions through the atmosphere by ongoing evaporation and deposition^3^.

Fenpropathrin (FNP) is a widely employed pesticide controlling of pests like mites and insects on diverse food items, including fruits and vegetables, grains, and tea. It is classified as a Category II pyrethroid insecticide and acaricide^4^. FNP can alter mitochondrial size by decreasing the expression level of dynein associated with mitochondria. It can generate highly reactive metabolites and reactive oxygen species (ROS) via FNP, which may induce oxidative stress. Furthermore, it modifies the gating kinetics of voltage-sensitive sodium channels, impairing neuronal function and causing acute neurotoxic consequences in insects and other organisms as the primary mode of action^5^. FNP may cause considerable hepatotoxicity and nephrotoxicity^6,7^. The liver is the principal organ impacted by many environmental contaminants since it functions as the central location for metabolic and detoxifying processes^8^. Additionally, some dietary pollutants are absorbed through the digestive tract and conveyed to the liver via the portal vein. The predominant route for FNP excretion is acknowledged to be via the kidneys. The kidney, the principal excretory organ in livestock, is essential to achieving homeostasis^6^.

Herbs have a long-standing history of promoting health and their potential for medication development. Herbal drugs derived from plants are increasingly employed to treat various disorders, including hepatic and renal problems, coronary artery disease, diabetes, high blood pressure, and cancer^9^. Investigating natural compounds with antioxidative properties that might avert or mitigate harmful changes would be particularly exciting^10^.

Elder, or *Sambucus nigra Linn*., is a member of the Adoxaceae family and a deciduous annual shrub or tiny tree. Europe, North Africa, and Asia are its native habitats. The plant possesses slender, dark green leaflets grouped oppositely, measuring approximately 10–30 cm long and developed through pinnate development. An inflorescence, produced between April and August, comprises multiple distinct fragrant white flowers featuring five petals and five stamens, releasing a delightful, strong fragrance^11^. *Sambucus nigra* has 154 components derived from the ethanol extract of its leaves. The substances include 72 flavonoids and their derivatives, 44 phenolics, 24 fatty acids, five organic acids, seven coumarins, one cyclitol, and one alcohol^5^. Elderberry leaves possess a significant concentration of polyphenols, mostly flavonoids like kaempferol and quercetin, and related compounds, including isoquercetin, rutin, astragalin, and myricetin. The leaves of elderberries include phenolic acids, including p-coumaric acid, chlorogenic acid, caffeic acid, neochlorogenic acid, and dicavoylquinic acids, in addition to amino acids and tocopherols^12^.

The study has two objectives. The main aim is to investigate the probable underlying mechanisms of hepatorenal injury associated with FNP. Secondly, the objective is to assess the efficacy of *Sambucus nigra* leaves extract in alleviating hepatorenal injury induced by FNP. The experiment was conducted over 60 days on rats, encompassing biochemical, histological, and molecular examinations.

## Materials and methods

### Chemical

The research employed pesticide formulations commercially obtained from Sumitomo Chemical Co., Ltd., Japan. We diluted the widely available insecticide Fenpropathrin (Danitol^®^ 20% EC) with corn oil to attain the requisite concentration of the active ingredient. In September 2022, two kilograms of *Sambucus nigra* foliage were procured from Morocco. The plant material was authenticated by comparison with the voucher specimens deposited at the Cairo University Herbarium (CAI), and identified as *Sambucus nigra L*. by Dr. Wafaa Amer, Professor of Plant Taxonomy and Flora (CAI). The specimen is registered under the species name following the Engler classification system, arranged alphabetically without a numerical code, and is available for reference upon request. The leaves were pulverized into a fine powder by a mechanical herb grinder. One kilogram of elderberry leaves underwent dehydration and was ground into a fine powder. Subsequently, the powder was immersed in 70% ethanol at ambient temperature for maceration. This process was repeated until more extraction proved impossible. Nashed et al. (2024) report that the leaves of *Sambucus nigra* yielded 286 g of dark green extract.

### Animals and animal grouping

Thirty male Wistar rats weighing between 160 and 200 grammes were procured from the animal facility of the Research Institute of Ophthalmology in Giza, Egypt. The rodents were fed a typical commercial pelleted diet and kept in plastic cages. And they had unlimited access to water. Prior to utilization, the individuals had a health evaluation and were acclimatized to the laboratory environment for 2 weeks. All experimental procedures were performed according to the Guide for the Care and Use of Laboratory Animals (NIH), the Animal Research: Reporting of in vivo Experiments (ARRIVE) guidelines, and approved by the Institutional Animal Care and Use Committee (IACUC) of the Faculty of Veterinary Medicine, Cairo University, following the Animal Use Protocol (Vet CU 13102024980).

Over 60 days, rats were randomly allocated into six groups (*n* = 5) and administered certain materials daily through oral gavage.

Group (1): Control group administered corn oil daily for 60 days.

Groups (2 and 3) were given SN (200 and 400 mg/kg/day), respectively, according to^13^.

Group (4) was given FNP at a dose of 4.7 mg/kg/day, equivalent to one-fifteenth of the LD_50_., according to^14^. 

Groups (5 and 6) were given both FNP and SN at the previously specified dosages.

### Sample collection

Upon concluding the experiment, blood samples were collected from the retro-orbital plexus. Clear serum samples, centrifuged for 20 min at 3000 rpm, were stored at −20 °C until utilized for biochemical analysis. The experimental animals were anesthetized via intraperitoneal injection of ketamine (90 mg/kg) and xylazine (10 mg/kg), then euthanized by cervical dislocation. The liver and kidneys were harvested from each animal. A segment of the sample tissue was preserved in sealed plastic bags at −80 °C to evaluate oxidative damage indicators and perform real-time PCR examination. The residual specimens were submerged in a 10% neutral-buffered formalin solution for histopathological and immunohistochemical analysis.

### Liver and kidney function

The manufacturer’s kits (Biodiagnostic, Cairo, Egypt) were used to measure the levels of ALT (alanine aminotransferase) (CAT. NO. AL 10 31), AST (aspartate aminotransferase) (CAT. NO. AS 10 61), ALP (alkaline phosphatase) (CAT. NO. AP 10 20), BUN (blood urea nitrogen) (CAT. NO. UR 21 10), and CRE (creatinine) (CAT. NO. CR 12 50) in serum specimens using a spectrophotometer (UNICO Instruments, UV-2100, USA).

### Oxidative stress evaluation

Liver and kidney samples were assessed for oxidative stress markers, specifically reduced glutathione (GSH) (CAT. NO. GR 25 11) and the lipid peroxidation biomarker malondialdehyde (MDA) (CAT. NO. MD 25 29). The evaluation was performed using the methodologies outlined by^15,16^ respectively. Commercially obtainable kits from Bio-diagnostics were utilized for this purpose. The liver and kidneys were perfused with a phosphate-buffered saline solution at a pH of 7.4, enhanced with 0.16 mg/ml of heparin. This perfusion method aimed to eliminate any presence of red blood cells and thrombi. The tissue samples were homogenized in a cold buffer solution containing 50 mM potassium phosphate and 1 mM EDTA at a volume of 5–10 ml per gram of tissue. Homogenization was conducted with a tissue homogenizer. The homogenate underwent centrifugation at 4,000 rpm for 15 min at 4 °C. The supernatant was preserved at −80 °C and analyzed using a spectrophotometer.

### Histopathological examination

The liver and kidney tissues specimens were gathered and fixed in 10% formalin in neutral buffered. They subsequently underwent normal processing in accordance with the protocol defined by^17^. The specimens were subjected to a quick cleaning with running water for several minutes, followed by dehydration with graded alcohol, xylene clearing, paraffin wax embedding, and sectioning into 4.5 μm slices. All slices were stained with haematoxylin and eosin and thereafter viewed blinded with an Olympus BX43 light microscope. Photomicrographs were obtained using an Olympus DP27 digital camera linked to the CellSens Dimensions program. The discovered lesions were evaluated independently among groups utilising the semiquantitative method delineated by^18^. Tissue alterations were categorised as mild, moderate, severe, or extensively severe according to the lesion extent per field on a grading system from 1 to 4, where (1) indicates mild changes and (4) denotes extensively severe changes.

### Immunohistochemical staining

Sections of liver and kidney from various experimental groups were treated with primary antibodies targeting casp-3 and iNOS (Abcam, Ltd.), diluted to a concentration of 1:200. Subsequently, the samples were incubated for the antigen-antibody immunological reaction utilising the secondary antibody, followed by counterstaining with haematoxylin. They were then viewed under an Olympus BX43 light microscope to determine the degree of immunopositivity from each group participating in the experiment.

### Quantitative RT-PCR for TNF-α and NF-_K_B genes

RNA extraction utilized TRIzol reagent (Invitrogen, USA), and RNA reverse transcription was executed with the PrimeScript RT reagent Kit (Takara, Japan). The quantitative reverse transcription polymerase chain reaction (qRT-PCR) was conducted utilizing the QuantStudio™ 5 Real-Time PCR System with SYBR Green (Thermo Fisher Scientific, USA). The thermal profile consisted of 95 °C for 10 s, 60 °C for 30 s, and 72 °C for 15 s, repeated for 40 cycles, preceded by an initial denaturation of 10 min at 95 °C and concluded with a final extension of 10 min at 72 °C. Subsequent to normalization against β-actin. The 2-ΔΔCt technique was employed to assess mRNA expression levels. The mRNA expression was standardized against that of β-actin. The primer set used in the study were shown in Table (1).

**Table 1:** The sequences of primers used for quantitative Real-time PCR.

Gene	Forward primer	Reverse primer
NF-κB	5′-GCTTTGCAAACCTGG GAATA-3′	5′-CAAGGTCAGAAT GCACCAGA-3′
TNF-α	5′-GGG GCC ACC ACG CTC TTC TGT-3′	5′-GCA AAT CGGCTG ACG GTG TGG-3′
β-actin	5′-CTGAGAGGGAAATCGTGCGT − 3′	5′-TTGTTGGCATAGAGGTCTTTA-3′

### Statistical analysis

The statistical study employed SPSS version 16.0 software created by SPSS Inc., based in Chicago, IL, USA. Findings were expressed as means ± SE (standard error). ANOVA (One-way analysis of variance) was employed to compare means among multiple groups, whereas an independent t-test was utilized to compare means between two groups. The threshold for statistical significance was defined as p-values < 0.05. Otherwise, the nonparametric data including histological scoring was analysed using Kruskal Wallis test followed by Mann-Whitney U test and presented as the median plus or minus the interquartile range (IQR).

## Results

### Liver and kidney function

**Fig. 1:**
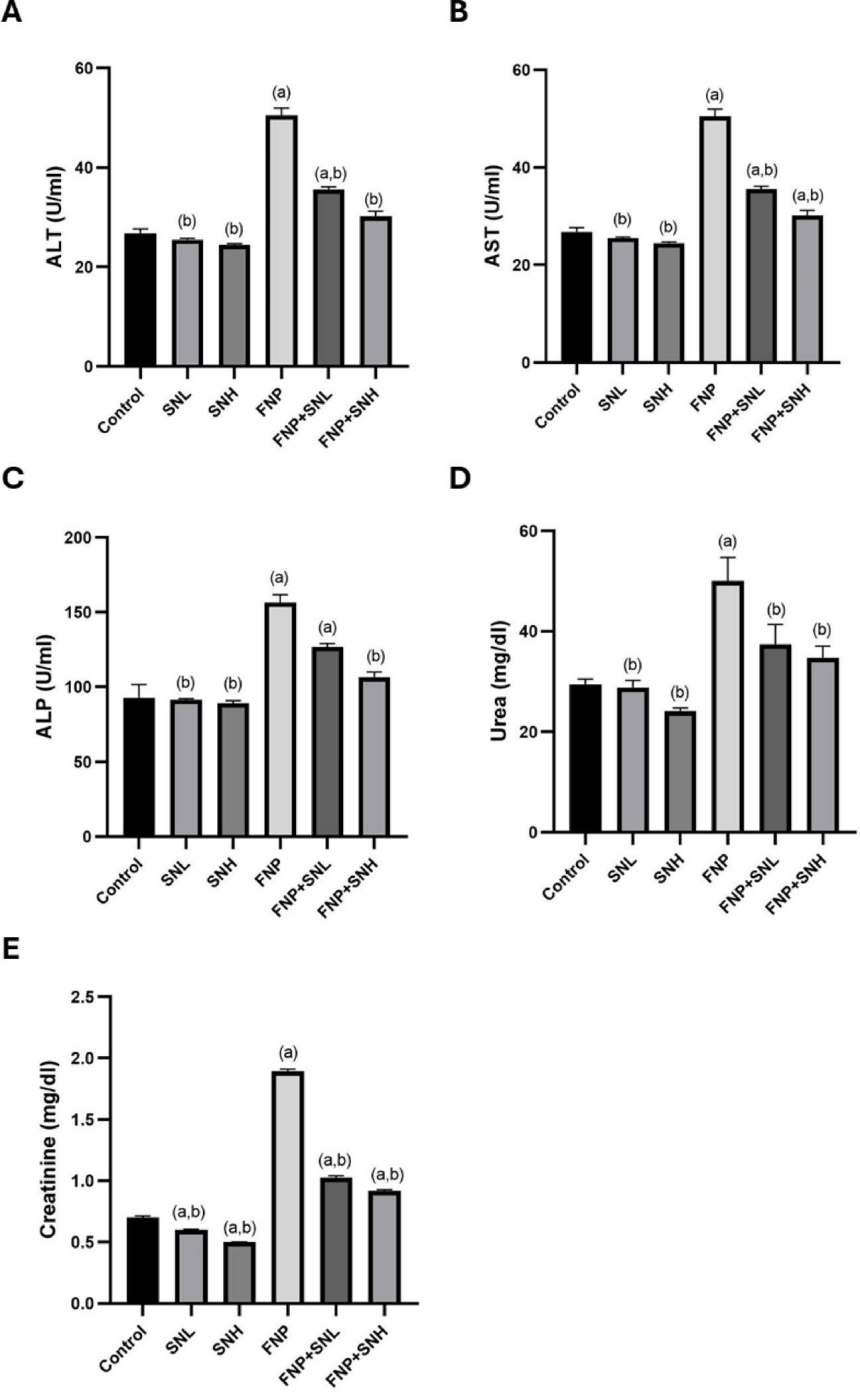
Liver and kidney function. (**A**) ALT, (**B**) AST, (**C**) ALP, (**D**) BUN, and (**E**) Creatinine. Data expressed as Mean ± SE. (a) represents a statistically significant variance from the equivalent Control group at *P* ≤ 0.05. (b) represents a statistically significant variance from the equivalent FNP group at *P* ≤ 0.05.

The group receiving FNP had significantly increased serum levels of ALT, AST, ALP, BUN, and creatinine relative to those in the control group. Nevertheless, the cohort administered FNP + SN demonstrated a considerable reduction in the previously mentioned indicators compared to the group that got only FNP **(**Fig. 1**).**

### Oxidative stress evaluation

**Fig. 2:**
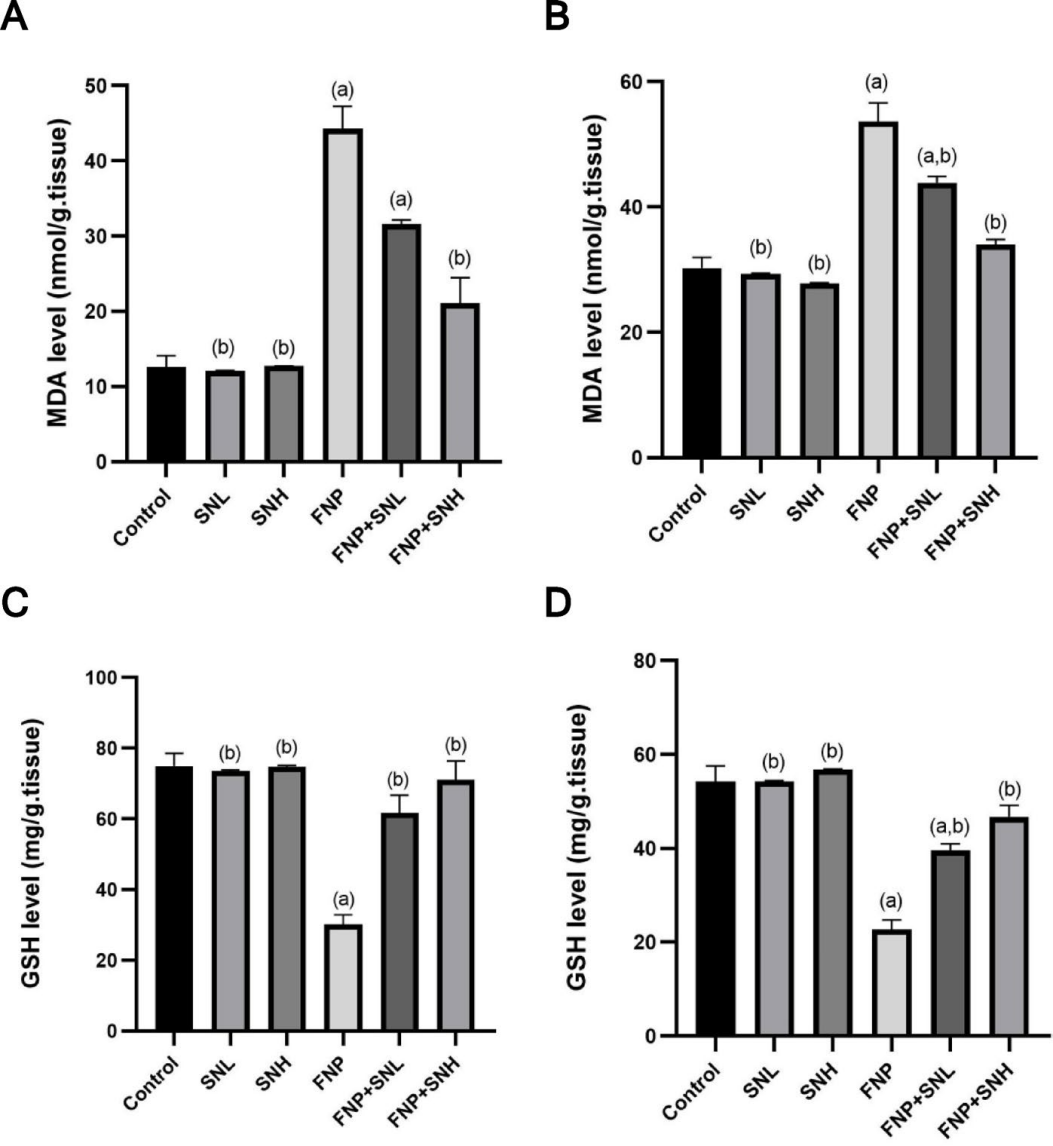
Hepato-renal oxidative stress evaluation. (**A**) Hepatic levels of MDA, (**B**) Renal levels of MDA, (**C**) Hepatic levels of GSH, and (**D**) Renal levels of GSH. Data expressed as Mean ± SE. (a) represents a statistically significant variance from the equivalent Control group at *P* ≤ 0.05. (b) represents a statistically significant variance from the equivalent FNP group at *P* ≤ 0.05.

Chronic and sustained consumption of Fenpropathrin exhibited detrimental effects on the balance between oxidants and antioxidants. This was demonstrated by a significant elevation in MDA levels and a reduction in GSH levels relative to the control group. Conversely, the coadministration of *Sambucus nigra* extract improved this balance by increasing GSH levels and decreasing MDA levels **(**Fig. 2**).**

### Histopathological examination

**Fig. 3:**
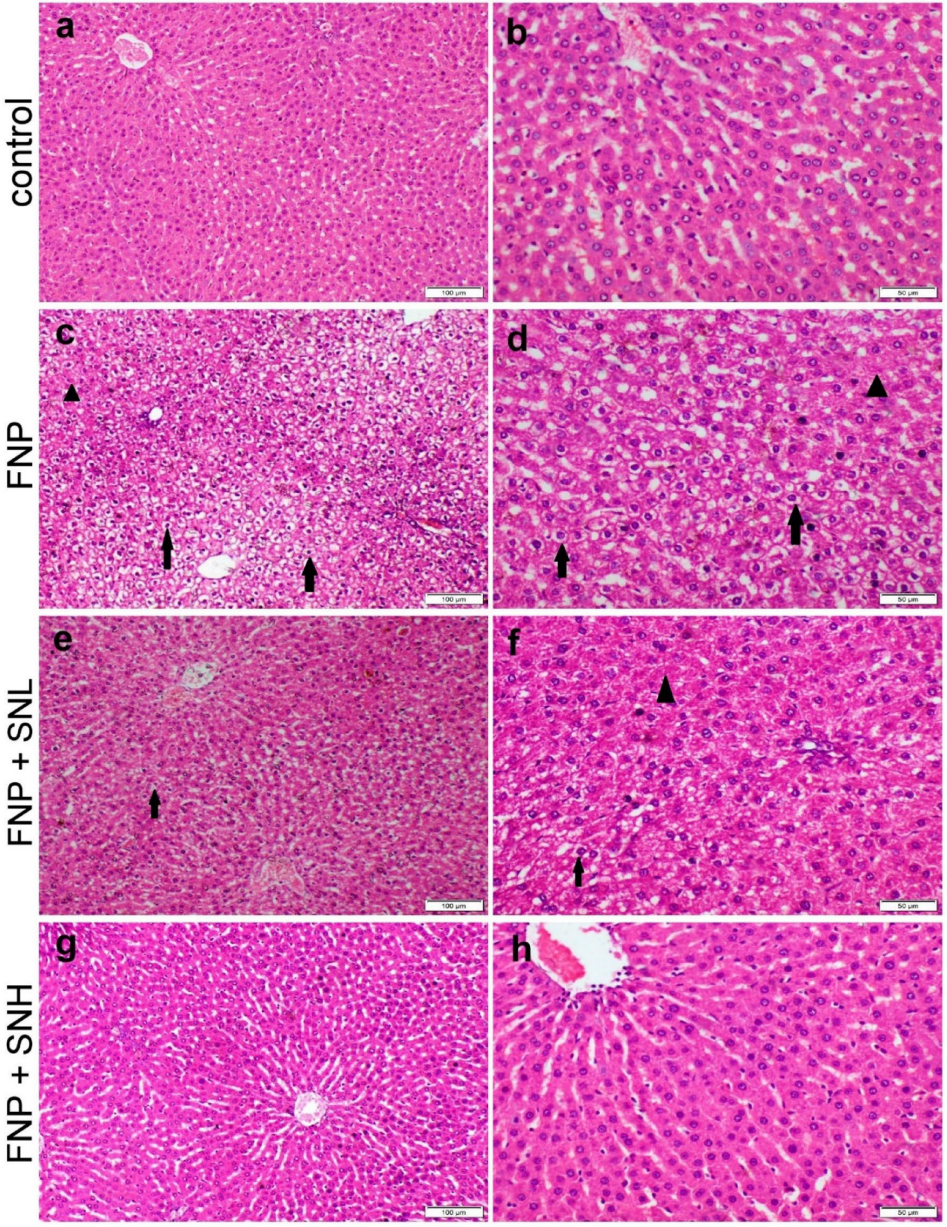
Histopathological appearance of H&E-stained liver sections obtained from various treatment groups. (**a**, **b**) control group showed normal histology. (**c**, **d**) FNP receiving group showed severe diffuse hepatocellular degeneration (arrow) and moderate necrosis (triangle). (**e**, **f**) group administrated low dose of SN with FNP showed moderate hepatocellular degeneration (arrow) and necrosis (triangle). (**g**, **h**) group administrated high dose of SN with FNP showed normal histological structure.

Liver sections from the control rats and those receiving SN at both doses showed normal histological architectures (Fig. 3a, b). While liver of rats receiving FNP showed severe diffuse hepatocellular vacuolar degeneration along with moderate hepatocellular necrosis (Fig. 3c, d). On the other hand, the liver of rats treated with SN at two doses showed varying degrees of improvement. Rats receiving low dose of SN with FNP demonstrated moderate hepatocellular vacuolization and necrosis (Fig. 3e, f), but those receiving high dose of SN with FNP showed normal (Fig. 3g, h).

**Fig. 4:**
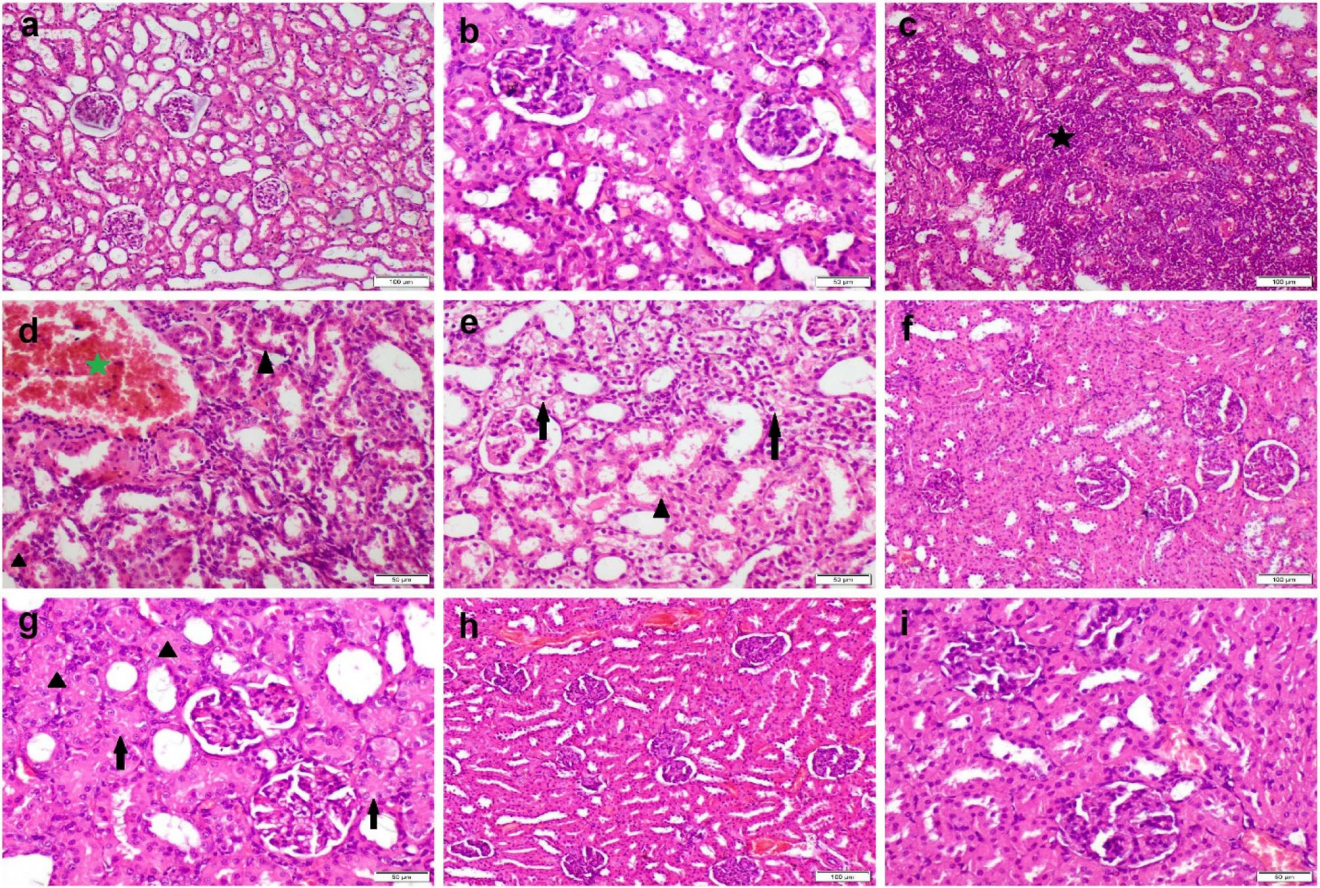
Histopathological appearance of H&E-stained kidney sections obtained from various treatment groups. (**a**, **b**) control group showed normal histology. (**c**-**e**) FNP receiving group showed severe tubular vacuolization (arrow) and necrosis (triangle), interstitial inflammatory cells infiltration (black star), and congestion (green star). (**f**, **g**) group administrated low dose of SN with FNP showed moderate tubular degeneration (arrow) and single cell necrosis (triangle). (**h**, **i**) group administrated high dose of SN with FNP showed normal histological structure.

The kidney section from the control rats and those receiving SN at both doses showed normal histological architectures (Fig. 4a, b). While kidneys of rats receiving FNP showed severe interstitial inflammatory cells infiltration (Fig. 4c) with extensive degeneration and necrosis of tubular epithelium (Fig. 4d, e). On the other hand, rats treated with SN at two doses showed varying degrees of improvement in kidney sections. Rats receiving low dose of SN with FNP demonstrated moderate tubular degeneration along with focal interstitial inflammatory cells infiltration (Fig. 4f, g), but those receiving high dose of SN with FNP showed normal histology (Fig. 4h, i).

The grading of the microscopic appearance for both liver and kidney tissues was recorded in Table (2) and showed a significant increase in the score of all parameters of hepatorenal alterations in FNP group compared with the control group. Otherwise, a significant decrease in such scores in the group cotreated with SN either at low or high doses compared with FNP group. The group cotreated with high SN dose had unsignificant differences in some parameters compared with the control group.

**Table 2:** Grading of hepatorenal histological alterations across groups.

	Control	SNL	SNH	FNP	FNP + SNL	FNP + SNH
Hepatic histological alterations						
HCD	0 ^a^	0 ^a^	0 ^a^	4 ^c^	1 ^b^	0 ^a^
HCN	0 ^a^	0 ^a^	0 ^a^	2 ^b^	0 ^a^	0 ^a^
Renal histological alterations						
TD	0 ^a^	0 ^a^	0 ^a^	4 ^d^	2 ^c^	1 ^b^
TN	0 ^a^	0 ^a^	0 ^a^	4 ^c^	1 ^b^	0 ^a^
Inflammation	0 ^a^	0 ^a^	0 ^a^	4 ^c^	1 ^b^	0 ^a^

Data expressed as median (*n* = 21 microscopic fields per group). Values carrying different subscript letters mean a significant difference between groups at *P* ≤ 0.05.

### Immunohistochemical staining

**Figs. 5:**
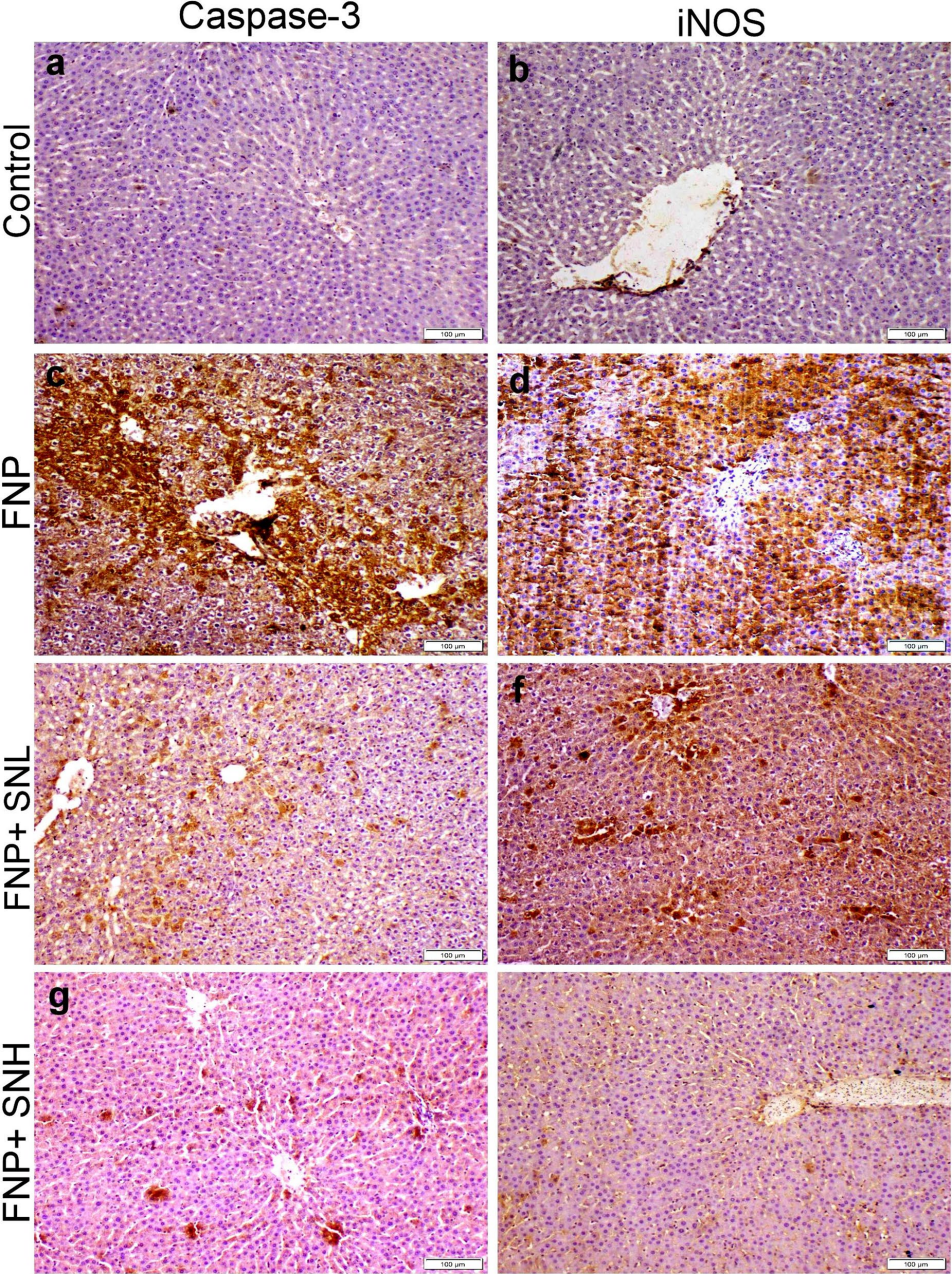
Caspase-3 and iNOS localization within hepatic tissue sections obtained from various groups. (**a** & **b**) control group showed normal baseline casp-3 and iNOS immunostaining, respectively. (**b**) FNP receiving group exhibit strong casp-3 and iNOS immunostaining, respectively. (**c**) group administrated low dose of SN with FNP showed moderate casp-3 and iNOS immunostaining, respectively. (**d**) group administrated high dose of SN with FNP showed weak casp-3 and iNOS immunostaining, respectively.

**Fig. 6:**
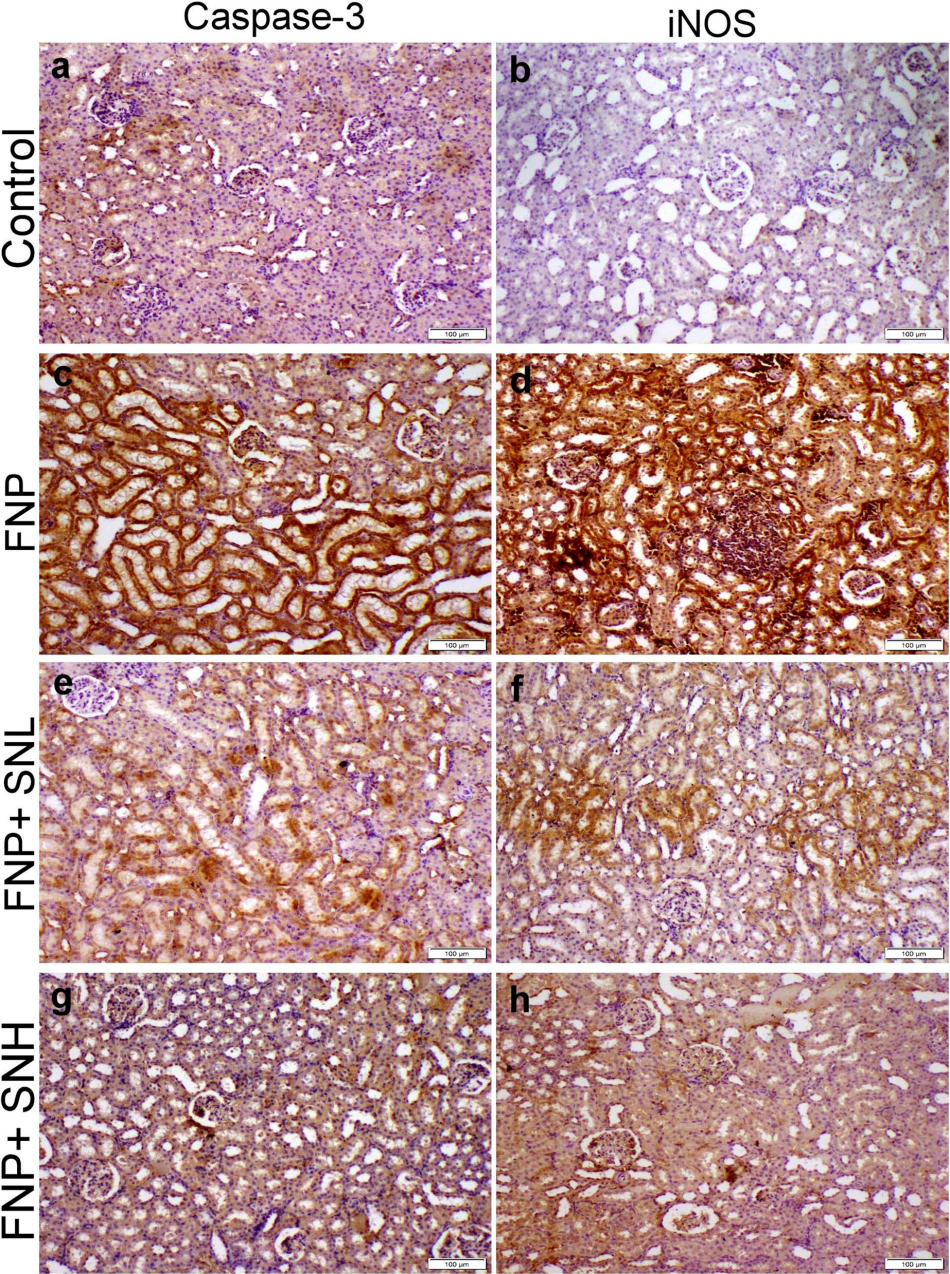
Caspase-3 and iNOS localization within kidney tissue sections obtained from various groups. (**a** & **b**) control group showed normal baseline casp-3 and iNOS immunostaining, respectively. (**b**) FNP receiving group exhibit strong casp-3 and iNOS immunostaining, respectively. (**c**) group administrated low dose of SN with FNP showed moderate casp-3 and iNOS immunostaining, respectively. (**d**) group administrated high dose of SN with FNP showed weak casp-3 and iNOS immunostaining, respectively.

Figures 5 and 6 demonstrate robust immunostaining expression of caspase-3 and inducible nitric oxide synthase in the liver and kidney sections of the FNP groups. Moreover, SN cotreatment significantly reduced the immunostaining intensity for both immunological markers in a dose-dependent manner.

### Quantitative RT-PCR for the genes TNF-α and NF-_K_B

**Fig. 7:**
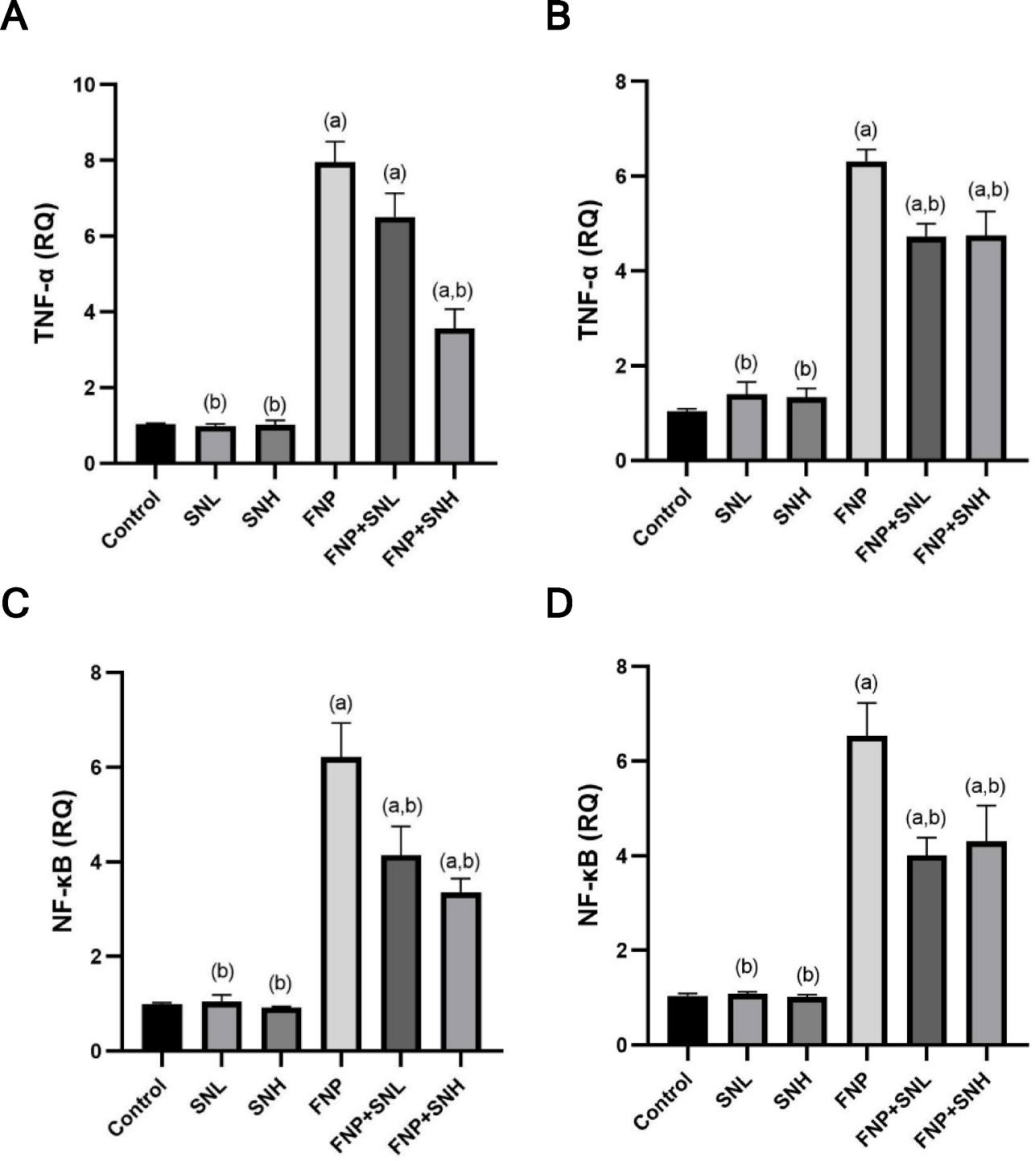
Levels of mRNA expression of TNF-α and NF-κB genes in both the liver and kidney. (**A**) mRNA levels of TNF-α in the liver, (**B** mRNA levels of TNF-α in the kidney, (**C**) mRNA levels of NF-_K_B in the liver, and (**D**) mRNA levels of NF-_K_B in the kidney. Data expressed as Mean ± SE. (**a**) represents a statistically significant variance from the equivalent Control group at *P* ≤ 0.05. (**b**) represents a statistically significant variance from the equivalent FNP group at *P* ≤ 0.05.

The group subjected to FNP exhibited a marked up-regulation of TNF-α and NF-κB mRNA levels in hepatic and renal tissues relative to the control group. The pre-treated groups receiving SN, either at low or high doses, had significant enhancement, evidenced by a marked reduction in TNF-α and NF-κB mRNA levels in the liver and kidneys relative to the group exposed to FNP toxicity. The remaining experimental groups displayed equivalent mRNA levels of the analysed genes in comparison to the control group **(**Fig. 7**).**

## Discussion

Environmental pollution is a globally important health problem, especially in impoverished countries^19^. Excessive pesticide exposure is acknowledged as a factor contributing to particular health issues in both people and livestock^1^. The current research aims to examine the potential negative impacts of fenpropathrin on the rat kidneys and liver. This will be accomplished by assessing biomarkers in the kidneys and liver, signs of oxidative stress, histological analysis, caspase-3, and iNOS immunological markers. Also, to understand the molecular toxicity of these pesticides in rats, we will measure the messenger RNA (mRNA) concentrations of the NF-κB and TNF-α genes. We will also look at how well *Sambucus nigra* protects rats from the harmful effects of fenpropathrin.

Experimental findings demonstrated that groups administered FNP exhibited increased serum levels of ALT, AST, and ALP. Hepatocytes often contain negligible amounts of aminotransferases, which are crucial for transamination processes in amino acid metabolism. Elevated aminotransferase levels in the blood signify hepatic membrane injury and leakage^20^. Moreover, ALP functions as a marker enzyme for the integrity of the hepatobiliary system; thus, increased blood levels of ALP indicate hepatobiliary damage^21^. The FNP therapy group exhibited higher quantities of blood urea and creatinine, signifying kidney impairment^22^. Serum creatinine and blood urea nitrogen (BUN) are frequently utilized as metrics for assessing renal function. Creatinine is filtered unimpeded in the glomerulus, with negligible amounts consistently discharged throughout the tubule^23^. BUN is a principal nitrogenous byproduct of proteins and amino acids; elevated BUN levels indicate compromised renal function^24^.

The findings of the current investigation indicated that FNP triggered hepatorenal oxidative stress injury, characterized by elevated concentrations of MDA and reduced GSH concentrations. These findings confirmed earlier research indicating that FNP may cause reactive oxygen species harm in the hepatic tissue of rats^25^. Furthermore, extended exposure to FNP induces alterations in inflammation and oxidative stress markers in the hepatic systems of rats^7^. Elevated MDA levels indicate augmented free radical generation, resulting in damage to the genome, destruction of proteins, lipids, and tissue damage, especially in the hepatocytes and renal^26^.

The oxidative stress evaluations indicated substantial pathological alterations in the hepatic and renal systems of rats subjected to FNP administration, which were caused by an overabundance of reactive oxygen species (ROS). Mitochondria play a crucial role in producing reactive oxygen species (ROS). The maintenance of normal physiological functions significantly depends on their structure and composition. Fenpropathrin may induce the generation of ROS by altering the MMP (mitochondrial membrane potential), compromising the mitochondrial complex’s functionality. Numerous investigations have demonstrated that increased ROS generation improves cell and mitochondrial, and the degradation of hepatocellular and renal tubular epithelial cells in the current investigation was explained by this mechanism. Moreover, oxidative stress raises cytosolic calcium levels by activating mitochondrial transition pores and causing mitochondrial malfunction^27^. The activation of several enzymes results in protein breakdown, oxidation of lipids, and destruction of DNA^28^. Those that induce further membrane damage and cellular demise through necrosis and apoptosis.

The development and preservation of tissues, along with the overall health of multicellular organisms, rely on the crucial and meticulously regulated process of cell death. It regulates cell growth and apoptosis, maintaining physiological equilibrium in mature organisms. This complex system functions during essential processes such as metamorphosis, embryogenesis, and tissue turnover, as well as in reaction to pathogenic threats. The two principal methods of cell death are programmed cell death (apoptosis) and necrosis. Apoptosis, regulated by signals, entails self-destruction in reaction to external or internal stimuli, serving a crucial function in preserving tissue homeostasis by removing redundant or damaged cells. While apoptosis is closely regulated, including essential components such as caspases, Bcl-2, Bax, and Bak, necrosis is a chaotic and uncontrolled form of cell death linked with pathological responses following severe cellular damage. Autophagic cell death and necroptosis are further planned forms that enhance the comprehension of these complex processes^29^.

Apoptosis is triggered by several stimuli, including the generation of ROS^30^. Multiple signaling mechanisms facilitate apoptosis, namely the intrinsic (mitochondrial) and extrinsic (death receptor) pathways. Initiator caspases (caspase-8 and − 9) and executioner caspases (caspase-3, −6, and − 7) are crucial in apoptotic signaling pathways^29^. The extrinsic route is activated by ligands such as tumor necrosis factor (TNF), which forms a death-inducing signaling complex (DISC) that subsequently activates caspase-8 and downstream caspases^31^.

Caspase-3, also known as CPP32 or apopain, is a protein produced by the CASP3 gene. It belongs to the endoproteases family, specifically cysteine-aspartic proteases. It utilizes a cysteine as the catalytic nucleophile in its active site to cleave target proteins at their aspartic acid residues. Caspase-3 is first produced as an immature zymogen (procaspase), requiring proteolysis to become activated. It comprises an N-terminal prodomain, a large subunit, and a small subunit. Indeed, after activation, this is the catalytic domain that is cleaved into a big and a small subunit, and the prodomain is eliminated^32^. Caspase-3 has been discovered as a critical mediator of apoptosis, triggered in apoptotic cells by both extrinsic (death ligands) and intrinsic (mitochondrial) routes^33^. During the terminal and execution phases of apoptosis, it is essential in delineating the array of substrates implicated in the distinctive morphological and biochemical characteristics found in apoptosis^34^. Additionally, nitric oxide (NO) is a recognized intercellular messenger that plays a multifaceted role in the immune system and inflammatory processes^35^. Inducible nitric oxide synthase (iNOS) is primarily associated with inflammatory conditions marked by elevated levels of nitric oxide (NO)^36^.

The current investigation demonstrated an increase of TNF-α and NF-κB mRNA levels in the liver and kidneys of rats exposed to FNP toxicity. Correlations have been identified between pesticide exposure and alterations in cytokine activity. Coremen et al. (2022)^37^ revealed that pesticide administration caused hepatotoxicity associated with pro-inflammatory cytokine production, which was subsequently elevated by oxidative stress. Moreover, many pesticides can induce ROS generation, resulting in oxidative stress and increased activation of the NF-κB pathway^38^. NF-κB, a transcription factor, is crucial in several biological processes, including cell adhesion, immunology, inflammatory conditions, differentiation, cell death, autophagy, division, and aging. Moreover, NF-κB governs the regulation of pro-inflammatory cytokines and can modulate inflammation associated with significant H2O2 generation. The disruption of NF-κB is implicated in several illnesses, such as asthma, cancer, cardiovascular disease, rheumatoid arthritis, neurological disorders, and inflammation^39^.

FNP stimulates substantial production of TNF-α and NO in macrophages, hence contributing to inflammatory responses, cytokine imbalance, and immunological dysregulation^40^. This study suggests that increased MDA concentrations in the liver and kidneys, together with heightened pro-inflammatory cytokine production, indicate that FNP may induce toxicity via the activation of the NF-κB signaling pathway during the chronic inflammatory phase.

The goal of our investigation was to examine the possible prophylactic impact of SN against hepato-renal injury generated by FNP. Several preventative properties of *Sambucus nigra* can be ascribed towards its phenolic bioactive components, including phenolic acids and flavonoids. These chemicals are prevalent in the foliage of *Sambucus nigra*^41^. The pretreatment of rats with SN in this study led to notable enhancements in all aforementioned toxicopathological parameters. The findings from the SN analysis consistently corroborated previous research indicating that *Sambucus nigra* possesses significant antioxidant activity associated with flavonoids. Quercetin demonstrates its antioxidant effects via multiple physiological routes. Primarily, it functions as an obvious hunter of free radicals, neutralizing the molecules and averting harm to cellular structures. Furthermore, quercetin amplifies the efficacy of pre-existing enzymes that produce antioxidants, including catalase, superoxide dismutase (SOD), and glutathione peroxidase, hence fortifying cellular defense against oxidative stress^42^.

The ingestion of *Sambucus nigra* successfully alleviated oxidative stress-induced damage in the liver and kidneys resulting from FNP. Moreover, the proactive action was validated histologically, demonstrating substantial enhancements in all histopathological parameters noted in hepatocyte and kidney tissue sections. The involvement of polyphenols in SN may help reduce tissue damage induced by FNP^43^. Moreover, SN can counteract the progression of degenerative lesions due to its potent antioxidant properties, enhanced biological effects of nitric oxide, and decreased inflammation^12^.

The therapeutic potential of SN is linked to its ability to diminish inflammation and suppress apoptosis. The results of our investigation indicated that the administration of SN to rats led to a substantial drop in the production of caspase-3 and iNOS proteins, along with a reduction in the levels of TNF-α and NF-κB proteins in both liver and kidney tissues. Numerous antioxidants, including tannins, phenols, and flavonoids included in *Sambucus nigra*, might directly or indirectly alleviate oxidative damage by suppressing the overproduction of free radicals^44^. The administration of SN to rats decreased caspase-3 and iNOS synthesis, hence alleviating oxidative stress damage and inflammatory responses. The prospective safeguarding of liver and renal tissues from pathological alterations may be ascribed to the anti-apoptotic, antioxidant, and anti-inflammatory characteristics of SN.

## Conclusion

Fenpropathrin can induce toxicity in the liver and renal tissues by harming cells via several routes. Our findings revealed the detrimental impact of FNP on hepatorenal tissues, evidenced by elevated blood levels of liver and kidney function biomarkers and a reduction in hepatorenal antioxidants. Furthermore, FNP induced several pathological damages in the hepatocytes and kidneys and stimulated the NF-κB/TNF-α pathway. The current study’s findings clearly indicate that *Sambucus nigra* extract (SN) can induce a dose-dependent enhancement in hepatorenal protection against fenpropathrin. Thus, the SN and its derivatives, as a powerful bioactive resource for the advancement of superior pharmaceuticals, may serve as an antidote, and their excellent tolerance would significantly mitigate the risk of overdose.

## Data Availability

Data is provided within the manuscript.
